# Microstructure imaging in patients undergoing evaluation for epilepsy surgery or low‐grade glioma: Clinical utility of a novel diffusion MRI method

**DOI:** 10.1002/epi4.70244

**Published:** 2026-03-06

**Authors:** Irena Grubor, Kristina Serednicka, Cornelia Säll, Maria Compagno Strandberg, Markus Nilsson, Johan Bengzon

**Affiliations:** ^1^ Division of Neurosurgery, Skane University Hospital and Department of Clinical Sciences Lund University Lund Sweden; ^2^ Department of Diagnostic Radiology, Skane University Hospital and Department of Clinical Sciences Lund Lund University Lund Sweden; ^3^ Medical Radiation Physics, Department of Clinical Sciences Lund Lund University Lund Sweden; ^4^ Diagnostic Radiology, Department of Clinical Sciences Lund Lund University Lund Sweden; ^5^ Division of Neurology, Skane University Hospital and Department of Clinical Sciences Lund Lund University Lund Sweden

**Keywords:** diffusion weighted imaging, DTI, focal cortical dysplasia, malformations of cortical development, mean diffusivity

## Abstract

**Objective:**

To investigate a recently developed MRI technique for mapping the Mean Diffusivity of Tissue (MDT), which improves diffusion MRI imaging of the cerebral cortex by reducing partial volume effects from cerebrospinal fluid (CSF). As cortical lesions are common in patients with focal epilepsy, we explored the clinical value of MDT imaging in patients with drug resistant epilepsy and low‐grade glioma.

**Method:**

This is a prospective study in which 18 patients undergoing evaluation for epilepsy surgery and five patients undergoing evaluation for low‐grade glioma were recruited. Patients underwent conventional MRI and MDT imaging. The images were analyzed by a neuroradiologist who visually assessed the images using a lesion visibility score.

**Results:**

Elevated MDT coincided with suspected FLAIR‐positive FCD lesions in the majority of cases (5 out of 8 lesions). Ulegyria was also MDT‐positive, but heterotopias were not. MDT did not detect abnormalities in epilepsy surgery cases previously deemed MRI‐negative. In low‐grade gliomas, MDT and FLAIR findings were co‐localized; however, the MDT abnormalities were more conspicuous, and tumor‐associated signals were more distinct relative to FLAIR. MDT was helpful in visual demarcation of ischemia and glioma tumor‐associated signals in postoperative imaging.

**Significance:**

MDT frequently aligns with the FLAIR signal, but MDT is visually more prominent. In the presurgical evaluation of epilepsy patients, it contributes to supporting existing or ambiguous findings. The very pronounced MDT signal in low‐grade gliomas might have the potential to be a visual aid in the diagnosis, treatment, and follow‐up of low‐grade gliomas.

**Plain Language Summary:**

This is a pilot study that investigates a newly developed MRI technique called MDT imaging. The study shows that MDT helps identify brain lesions in patients with epilepsy. Finding brain lesions can improve the success of epilepsy surgery and help patients become seizure‐free. MDT imaging also helps visualize tumors in low‐grade glioma patients, which could improve surgical planning. These early findings suggest that MDT could be an additional useful tool for imaging in epilepsy surgery and low‐grade glioma patients, but more research is required to confirm its benefits.

**Key points**

- MDT MRI is a novel diffusion imaging technique developed to improve imaging of the cerebral cortex.
- MDT MRI supported the visual assessment of microstructural alterations in patients undergoing evaluation for epilepsy surgery.
- MDT and FLAIR alterations co‐localized in the majority of FCD cases.
- In low‐grade gliomas, there was a very strong MDT signal that closely corresponded with the FLAIR signal.
- MDT was helpful in visual demarcation of ischemia and glioma tumor‐associated signals in postoperative imaging.

## 1 INTRODUCTION

Malformations of cortical development, especially focal cortical dysplasia (FCD), are common pathologies in epilepsy surgery, but can be difficult to visualize with MRI. Accurate presurgical detection and delineation of epileptogenic lesions on MRI are critical, as the seizure outcome in epilepsy surgery patients with structural MRI lesions is significantly better compared to MRI‐negative patients.^1^ The difficulty in identifying lesions such as FCD underscores the need to improve imaging for epilepsy surgery.

Diffusion‐Weighted Magnetic Resonance Imaging (DWI) is a technique with many clinical applications for the diagnosis and monitoring of conditions such as stroke, infections, and brain tumors.^2^ The technique uses the random motion of water molecules in tissue to indirectly map the tissue microstructure. Among analysis methods, Diffusion tensor imaging (DTI) provides maps of fractional anisotropy (FA) and mean diffusivity (MD), which reflect the integrity and coherence of white matter fiber bundles and cellular density, respectively. Although DWI is not included in the HARNESS protocol (Harmonized Neuroimaging of Epilepsy Structural Sequences), DTI is still used in some epilepsy surgery centers as part of the presurgical evaluation for delineation of functional tracts, and DWI is used in selected cases for differential diagnostics of structural lesions.^3^ However, DTI suffers from well‐known limitations.^4^ Among the most widely recognized problems are crossing fibers, which can lead to low FA values even in regions with healthy white matter. Another important but less recognized problem is that of partial volume effects, which occur when a voxel captures signals from multiple tissues or regions, such as the cortex and cerebrospinal fluid (CSF). This is common as the imaging voxels are not much smaller than the thickness of the cortex. Changes in cortical MD could therefore reflect either cortical thinning with increased contributions from CSF or changes to the cortical diffusion itself.^5^ This inherent problem limits the use of diffusion MRI when assessing microstructural alterations in cortex.

In this study, we explore the utility of a recently developed approach for mapping the Mean Diffusivity of Tissue (MDT) unconfounded by partial volume effects with CSF.^6^ This is achieved by quantifying the MD in a high b‐value range, where the signal from CSF is suppressed to below 1%. Such conditions would normally cause partial volume effects with white matter or from the rectified noise floor, but here these effects are suppressed using spherical tensor encoding and enhanced signal‐to‐noise ratios by super resolution.^6^ By reducing partial volume effects, MDT could potentially improve imaging of cortical areas, and in this work, we aimed to explore its potential value in a clinical setting. The MDT method relies on spherical tensor encoding,^7^ which requires a specific diffusion MRI pulse sequence. Our hypothesis was that the approach could improve the detection of microstructural alterations relevant to epilepsy. We performed MDT imaging in drug‐resistant epilepsy patients undergoing epilepsy surgery evaluation, as well as in patients with suspected low‐grade glioma, as this is a patient group with a particularly high prevalence of epilepsy.

## 2 MATERIALS AND METHODS

In this prospective study, 18 patients undergoing evaluation for epilepsy surgery and five patients undergoing evaluation for low‐grade glioma were recruited between 2023 and 2025 at Skane University Hospital's Department of Neurology in Sweden. As this was a pilot study, the inclusion criteria were broad. We included MRI‐negative patients, patients with ambiguous findings on MRI, patients who had previously undergone epilepsy surgery but did not achieve seizure freedom and patients with radiological findings consistent with malformations of cortical development. Inclusion criteria for tumor patients were radiological suspicion of low‐grade glioma, with or without a seizure history. Exclusion criteria included pregnancy, Vagus Nerve Stimulator, or any contraindication to MRI. All patients, except for two of the suspected low‐grade glioma patients, had epilepsy and were on anti‐seizure medication. In total, 15 females and 8 males, with a median age of 33 years (min 10, max 58) at the time of examination, were recruited. Three patients were under 18 years. A healthy volunteer acquired in a separate study using the same protocol was also included here as a reference.^6^ The study was approved by the Swedish Ethical Review Authority. All subjects gave written informed consent according to the recommendations of the Declaration of Helsinki.

### 2.1 MRI acquisition, reconstruction, and processing

MRI was performed using a Siemens 3T scanner (MAGNETOM Prisma, Siemens Healthineers, Germany) using a 20‐channel head and neck coil. Morphological imaging was performed by acquiring T1‐weighted MPRAGE data as well as FLAIR images, in accordance with the HARNESS protocol. The resolution was 1 mm isotropic for the T1‐weighted MPRAGE scans. The FLAIR resolution depended on where the patient had their clinical MRI and was either 1 mm isotropic or 1 mm in plane but with thicker slices.

In addition, a research sequence for free waveform encoding (FWF) was used to acquire data with spherical tensor encoding (*b*‐values of 0, 1500, and 2500 s/mm^2^ in 1, 16, and 24 repetitions), using an echo time of 110 ms and a repetition time of 2100 ms.^8^ The diffusion‐encoding waveforms were optimized using the NOW‐toolbox, minimizing Maxwell terms.^9^ Echo‐planar imaging (EPI) was used, with an in‐plane voxel size of 1.53 × 1.53 mm^2^ and a slice thickness of 5.2 mm (partial Fourier 0.75, GRAPPA factor 2, SMS factor of 2). The imaging stack was acquired in six rotations around the anterior–posterior phase‐encoding axis to enable the reconstruction of high‐resolution images with isotropic 1.53 mm voxels using the super‐resolution reconstruction approach described previously.^10^ Prior to the super‐resolution step, denoising, averaging, and distortion correction were applied, using additional data acquired with the opposite polarity of the phase‐encoding direction.^10^, ^11^, ^12^, ^13^ Finally, we computed a parameter referred to as the MDT as

$$\mathrm{MD}=-\frac{\log\left(s\left(b_{2}\right)\right)-\log\left(s\left(b_{1}\right)\right)}{b_{2}-b_{1}}$$

where *b*_1_ = 1500 s/mm^2^ and *b*_2_ = 2500 s/mm^2^, in analogy with how the ADC is computed from conventional diffusion MRI using different *b*‐values.^2^, ^6^ The resulting MDT maps were co‐registered with the T1‐weighted data as well as resampled, and presented both in their original form and masked by a cortical mask obtained from running Synthseg on the T1‐weighted images.^14^ The acquisition time for MDT was approximately 1.5 min per rotation, giving a total acquisition time of approximately 9 min.

The MR images were reviewed by a neuroradiologist with expertise in epilepsy surgery, who had access to clinical data. The neuroradiologist compared signal intensity differences across images for each patient. The visual analysis was qualitative. Lesion visibility intensity was assessed using a lesion visibility score from 0 to 4, where 0 indicated no visible lesion, 1 indicated poorly visible, 2 indicated moderately visible, 3 indicated clearly visible, and 4 indicated highly conspicuous.

## 3 RESULTS

### 3.1 Normal findings

**FIGURE 1:**
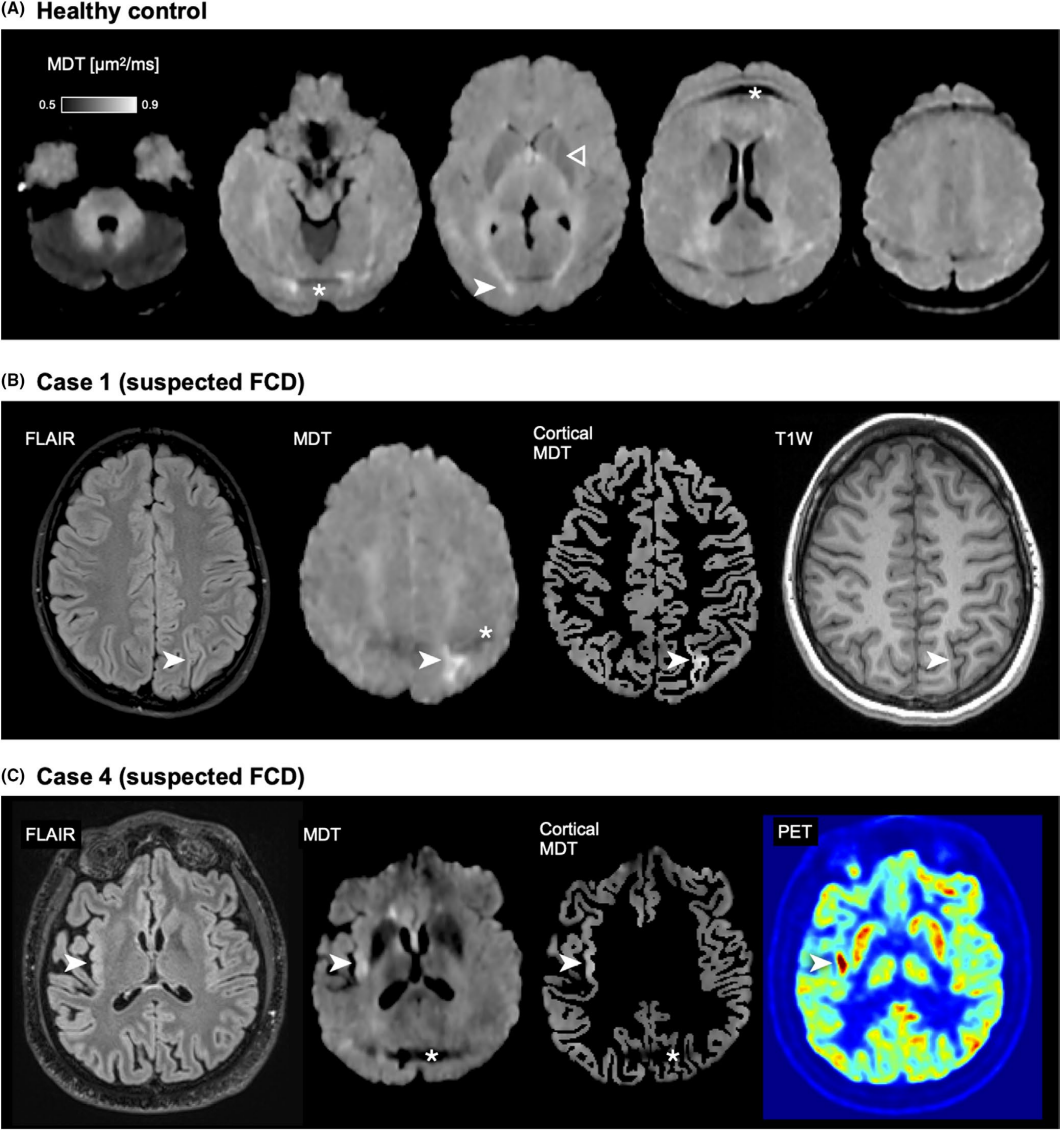
MDT patterns in a healthy participant and suspected focal cortical dysplasias (FCD). Panel A shows characteristic MDT signal features in a healthy brain. Hyperintensities are observed adjacent to the posterior horns coalescing with white matter tracts (white arrowheads), while the basal ganglia appear hypointense (hollow arrowhead). Fat artifacts appear as dark bands, for example, in the cerebellum and supratentorial regions (asterisk). The standard MDT color scale used in this work is shown. Panel B shows a suspected FCD (case 1). The lesion in the left parietal cortex is barely visible on transversal FLAIR and T1W. In contrast, it appeared conspicuous on MDT with standard windowing (white arrowheads). Application of a cortical mask confirmed that the MDT abnormality originates from the cortex. Panel C shows another suspected FCD (case 4). Increased signal on FLAIR and MDT in the right posterior insula (white arrowheads) was colocalized with a hypermetabolic region on PET. Cortical masking again demonstrated that a major component of the MDT intensity is cortical. Asterisks mark fat artifacts (B, C).

**FIGURE 2:**
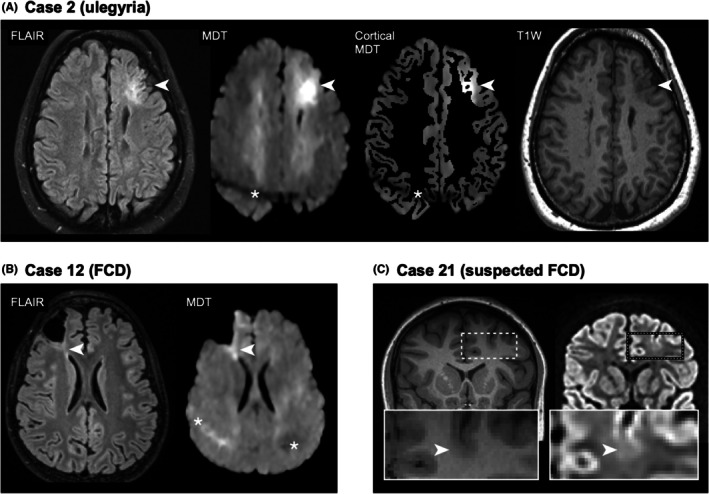
Ulegyria and focal cortical dysplasias (FCD). Panel A shows case 2 with ulegyria in the left frontal lobe. Hyperintensity is seen on both FLAIR and MDT involving the cortex and subcortex (white arrowheads). Cortical MDT confirmed cortical origin, and T1W showed apparent cortical thickening. An asterisk marks fat artifacts. Panel B shows case 12, a previously resected FCD. A transmantle sign in the right frontal lobe is visible on both FLAIR and MDT (white arrowheads). The overlying cortex had been resected, leaving no evident cortical component, though a mild signal increase was observed at the resection margin. Asterisks mark fat artifacts. Panel C shows case 21 with a bottom‐of‐sulcus dysplasia in the left frontal lobe (white arrowheads). The lesion was positive on FLAIR but negative on MDT. Cortical disruption was appreciable on high *b*‐value DWI (spherical encoding, *b* = 2500 s/mm^2^, white arrowheads), and cortical thickening was confirmed on T1W.

Figure 1A shows MDT images in a healthy control. A high MDT signal is interpreted as increased diffusion, whereas a low signal means reduced diffusion. White and gray matter were isointense in MDT, and CSF signal was dark. Structures with a high iron load, such as the globus pallidus, putamen, and substantia nigra, also appeared dark in the MDT image. Increased MDT was found adjacent to the posterior horns of the ventricles, and this “posterior capping” was present in all patients. These areas were coalescent with symmetrical bilateral diffuse posterior white matter hyperintensities on FLAIR, corresponding to the localization of the posterior white matter fiber bundles. Increased MDT was also found anteriorly and adjacent to the frontal horns of the lateral ventricles in 15 of the 23 patients (“anterior capping hyperintensities”). Some patients exhibited unspecific focal subcortical white matter hyperintensities on MDT, which correlated with FLAIR lesions. Symmetrical, easily recognizable artifacts from fat signals were frequently observed supratentorially and infratentorially on the MDT map (Figures 1 and 2). Hyperintense MDT was observed in near air‐tissue interfaces (sinuses or skull base). Increased MDT was also occasionally observed in localized areas where there was increased CSF, as in widened sulci.

### 3.2 Patients undergoing evaluation for epilepsy surgery

Of the 18 patients included, seven were classified as having FCD based on MRI findings, three of whom had previously undergone resections (Table 1, Table S1). Two patients presented with subependymal heterotopias, one of whom also had ulegyria, and a third patient presented with a hypothalamic hamartoma and polymicrogyria. Eight patients were MRI‐negative. Among the seven patients with FCD, MDT showed moderately or clearly visible lesions in two of the five non‐resected cases and in all three post‐resection cases. On conventional MRI (FLAIR), lesion visibility ranged from poor to clear. MDT showed higher visibility than FLAIR in three lesions, equal visibility in two, and did not detect three lesions visible on FLAIR. In the two heterotopia cases, MDT did not reveal any visible abnormalities, whereas all conventional sequences did (Figure S1). In the group with other malformations, the ulegyria lesion was clearly visible on MDT and poorly to moderately visible on the other sequences. The polymicrogyria and hypothalamic hamartoma were not visible on MDT but were visible on conventional imaging (Figure S1). Among the eight patients who were negative on conventional MRI, none had clinically relevant lesions on MDT. A meningioma was not detected on MDT but was moderately visible with conventional MRI. As the presurgical epilepsy evaluation findings were not concordant with the meningioma, it was considered incidental, and the patient was classified as MRI‐negative.

**TABLE 1:** Demographics and radiological characteristics of epilepsy surgery cases.

Case	Age	Age at seizure onset	Sex	Lesion location	MRI					
					MDT	DWI	ADC	FLAIR	T1	T2
Focal cortical dysplasia										
1	22	13	F	Parietal L	3	0	0	2	1	2
4	31	<10	M	Insula R	2	0	0	1	1	0
5	40	15	F	Temporal R	0	0	0	1	1	1
8	33	<1	F	Temporal R	0	0	0	2	1	1
21	17	2	F	Frontal L	0	0	0	1	1	2
FCD cases with previous resections										
3	46	26	F	Frontal resection cavity L, FCD remnant	2	0	1	2	1	2
8	33	<1	F	Parietal resection cavity R, FCD remnant	3	0	1	3	2	2
12	10	5	F	Frontal resection cavity R, transmantle remnant	2	0	0	1	0	1
Heterotopias										
2	36	<1	F	Subependymal bilat	0	2	2	1	2	2
7	55	21	M	Subependymal bilat	0	2	2	1	2	2
Other malformations										
2	36	<1	F	Ulegyria, frontal L	3	1	1	2	2	2
19	45	11	F	Polymicrogyria, R frontal	0	2	0	2	3	2
				Hypothalamic hamartoma	0	0	0	1	2	2
MRI‐negative										
6	32	4	F		0	0	0	0	0	0
9	25	8	M		0	0	0	0	0	0
10	43	29	M		0	0	0	0	0	0
13	48	44	F		0	0	0	0	0	0
14	14	6	F		0	0	0	0	0	0
18	21	14	F		0	0	0	0	0	0
22	19	9	M		0	0	0	0	0	0
11	37	34	F	Meningioma frontal L	0	2	1	1	2	2

*Note*: Age and age at seizure onset is presented in years. Sex F: female; M: male. MRI: L: left; R: right. Lesion visibility intensity assessment: 0: no visible lesion; 1: poorly visible; 2: moderately visible; 3: clearly visible, and 4: highly conspicuous.

Figure 1 shows MDT maps from a healthy control and two FCD cases (1 and 4) with moderately to clearly visible lesions on MDT. A cortical mask was applied to isolate cortical contributions to the MDT signal, revealing prominent cortical hyperintensities in both cases. Case 1, with a suspected FCD in the parietal cortex, showed clear abnormalities on both MDT and FLAIR (Figure 1B). Case 4, who experienced focal non‐motor seizures daily, had a suspected lesion in the posterior insula that demonstrated a strong MDT hyperintensity but only a subtle FLAIR abnormality (Figure 1C). In this case, the MDT signal was more conspicuous than FLAIR and closely matched a hypermetabolic area identified on a prior PET scan. This patient subsequently underwent laser ablation targeting this lesion. At the one‐year follow‐up, the patient had transitioned from an average of seven focal non‐motor seizures per day to seizure freedom with auras only (ILAE class 2). These cases illustrate how MDT may enhance the detection and delineation of cortical dysplasia, even in regions with subtle findings on conventional MRI.

Figure 2 shows results from cases with ulegyria and FCDs (cases 2, 12, and 21). In case 2, the ulegyria was clearly hyperintense on MDT both cortically and subcortically (Figure 2A). It was also clearly visible on FLAIR and T1W. In case 12, preoperative imaging without MDT displayed a clearly visible frontal FLAIR‐positive FCD with an adjacent transmantle sign (Figure 2B). After surgery, the patient was not seizure‐free. Postoperative imaging included MDT, and neither this nor conventional MRI revealed any FCD remnants in the resected cortical area. However, both MDT and FLAIR were positive for the remaining transmantle abnormality and demonstrated increased signal at the resection margins (Figure 2B). This pattern of an increased MDT and FLAIR signal immediately adjacent to the resection margins, was evident in patients with relatively recent resective surgeries (case 3, 4 months; case 12, 9 months) as well as in a patient who had undergone surgery 18 years prior (case 8). Increased MDT signal, as well as FLAIR signal, was also evident in trajectories after previous intracranial electrode implantations (cases 5 and 6). In case 21, conventional MRI demonstrated a suspected bottom‐of‐sulcus dysplasia, whereas MDT imaging was negative (Figure S1). However, case 21 also presented with an unexpected finding; the high *b*‐value DWI (*b* = 2500 s/mm^2^) images revealed a distinct cortical disruption at the bottom of the sulcus (Figure 2C). A similar cortical disruption associated with FCD was also observed in case 4.

Apart from the cases shown in the figures, there are two other noteworthy cases. Case 3 underwent resection of a visible FCD in the frontal lobe but was not seizure‐free after the first surgery. Postoperative MRI showed a clear FLAIR and MDT‐positive FCD remnant. After a reoperation, the resected tissue was histopathologically confirmed as an FCD type IIa, and the patient was seizure‐free at 1‐year follow‐up (ILAE class 1). Case 8 had undergone previous surgery for FCD in the parietal lobe but was not seizure‐free. Imaging demonstrated a suspected remnant adjacent to the resection cavity, positive on both MDT and FLAIR. The same patient also had a suspected FCD in the temporal lobe that was not MDT‐positive (Figure S1).

### 3.3 Tumor patients

Five patients with radiologically suspected low‐grade glioma were included (Table 2). All patients underwent surgical procedures with histological examination, and the histopathological diagnoses in the three resection cases were: low‐grade astrocytoma grade II‐III (case 16), oligodendroglioma grade II (case 17), and high‐grade diffuse astrocytoma grade IV (case 23). Two of the confirmed tumor cases (cases 16 and 23) had epilepsy and anti‐seizure medication. The biopsy cases revealed normal histology.

**TABLE 2:** Demographics, radiological features, and histology of suspected low‐grade glioma cases.

Case	Age	Epilepsy	Sex	Lesion location	MRI						Histology
					MDT	DWI	ADC	FLAIR	T1	T2	
Resections											
16	24	Yes	F	Temporal R	4	1	2	3	2	3	Astro grade II‐III
17	58	No	M	Frontal L	4	1	2	3	2	3	Oligo grade II
23	49	Yes	M	Mulitfocal R	4	1	2	3	2	3	High‐grade Astro IV
Biopsies											
15	58	No	M	Amygdala L	3	0	0	2	1	2	Normal
20	28	Yes	F	Amygdala R	1	0	0	1	1	1	Normal

*Note*: Age is presented in years. Sex: F: female; M: male. MRI: L: left; R: right. Lesion visibility intensity assessment: 0: no visible lesion; 1: poorly visible; 2: moderately visible; 3: clearly visible, and 4: highly conspicuous. Histology: Astro: astrocytoma; Oligo: oligodendroglioma. Case 16: Astrocytoma grades II–III, isocitrate dehydrogenase (IDH) mutation. Case 17: Oligodendroglioma grade II, IDH mutation, 1p/19q co‐deletion. Case 23: High‐grade diffuse astrocytoma, grade IV, IDH wild type.

**FIGURE 3:**
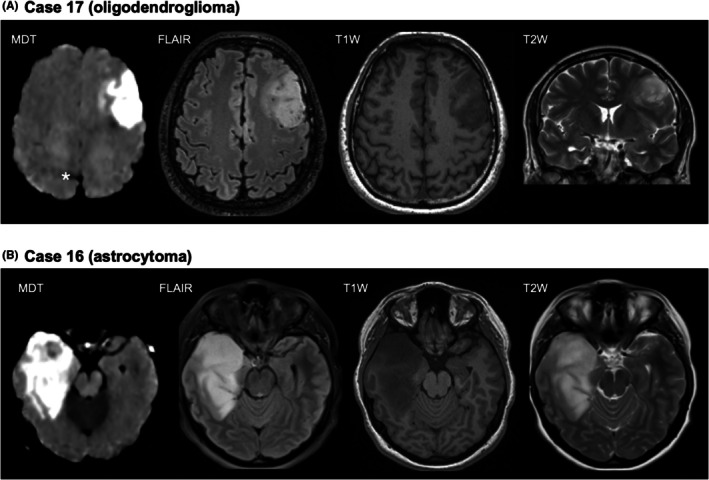
Oligodendroglioma and astrocytoma. Panel A shows case 17 with a grade II oligodendroglioma. MDT and FLAIR both demonstrated strong hyperintensity, with sharper delineation on MDT. T1W showed hypointensity and T2W hyperintensity. Panel B shows case 16 with a grade II–III astrocytoma. MDT demonstrated a strong hyperintensity closely following the FLAIR signal, again with sharper delineation compared to FLAIR. An asterisk marks a fat artifact. Again, T1W showed hypointensity and T2W hyperintensity in the same region.

**FIGURE 4:**
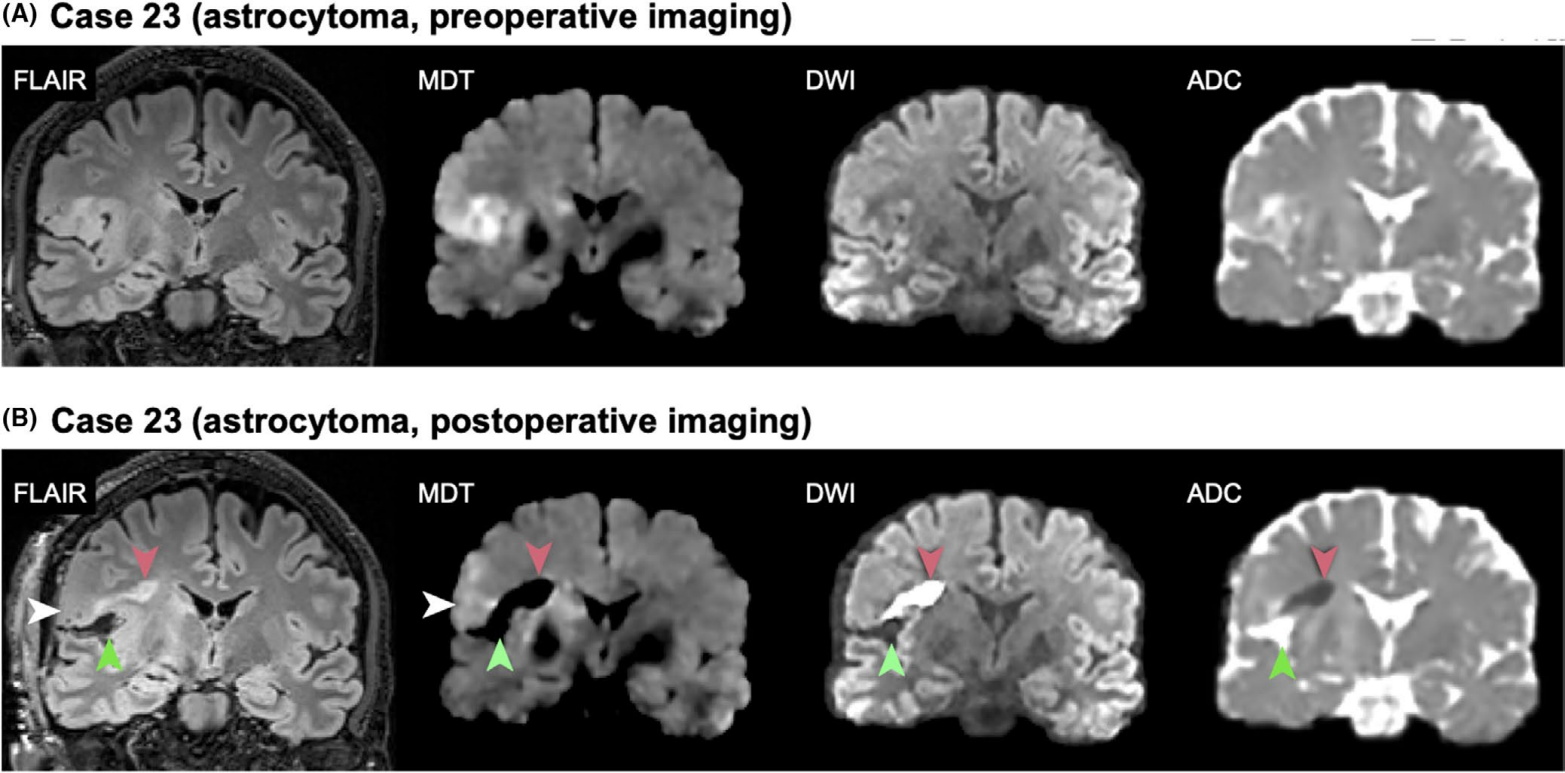
Pre‐ and postoperative imaging after resection of high‐grade diffuse astrocytoma. Panel A shows preoperative images from case 23 with multifocal grade IV astrocytoma involving the right frontal lobe, anterior insula, caudate nucleus, and mesencephalon. Tumor regions in the frontal lobe and insula appeared hyperintense on both FLAIR and MDT. Panel B shows postoperative imaging after resection of the frontal operculum and insula. Residual tumor was visible on FLAIR and MDT (white arrowhead). New ischemia was detected as reduced diffusion on MDT and ADC with corresponding hyperintensity on DWI, and faint hyperintensity on FLAIR (red arrowheads). CSF in the resection cavity was nulled on MDT (green arrowhead), making it indistinguishable from ischemia, although MDT still differentiated ischemia from tumor remnants within the same image. In contrast, FLAIR required correlation with diffusion imaging for reliable evaluation of tumor remnants.

Radiologically, none of the suspected low‐grade glioma cases displayed any distinct gadolinium contrast enhancement. In all cases, the regions of MDT hyperintensity colocalized with the FLAIR hyperintensity, but the MDT hyperintensity was unquestionably more conspicuous (Figure 3). While the hyperintense FLAIR regions had unclear margins, the MDT hyperintense regions were more sharply delineated and easier to identify in the glioma cases. As such, MDT appeared to offer improved clarity in the visual demarcation of the tumor‐associated signal (Figure 3). In case 23, MRI including MDT was performed both before resection and within 48 h postoperatively (Figure 4). Preoperatively the MDT hyperintensity matched the FLAIR hyperintensity except for a FLAIR‐positive area in the vicinity of the basal ganglia and the mesencephalon where hypoattenuated MDT signal from iron‐rich structures might have obscured the signal. Postoperatively, suspected tumor remnants were evident with both FLAIR and MDT. Ischemia was dark on the MDT map. There was no obvious hyperintensity in the resection margins.

## 4 DISCUSSION

To improve seizure outcomes in epilepsy surgery, additional radiological biomarkers are needed for better detection and delineation of structural alterations in the presurgical evaluation. In this study, we explored a novel diffusion imaging technique—so‐called MDT mapping—in epilepsy surgery and low‐grade glioma patients. The approach is distinguished from other diffusion MRI methods by its ability to suppress partial volume artifacts from CSF and white matter when imaging cortical microstructure. An advantage of MDT compared to MD‐mapping with DTI is that MDT enables a rapid visual assessment that reveals both cortical and subcortical alterations, as the CSF is effectively suppressed. By suppressing the signal from CSF, the MDT can be displayed with a tight window (0.5–0.9 μm^2^/ms), which increases sensitivity to subtle alterations in the parameter. Limiting the windowing range of regular MD or ADC to such a tight range would result in many oversaturated regions, which would reduce the readability of the map.

In this work, we found that MDT was valuable in detecting clinically relevant radiological changes in both epilepsy surgery and low‐grade glioma patients. Increased MDT signal corresponded to a suspected FLAIR‐positive FCD lesion in more than half of the lesions (5 out of 8 FCD lesions). There was no clear delineation of FCDs on the MDT map, but the signal corresponded to that seen on FLAIR. MDT detected a remaining subcortical transmantle alteration in a patient who had undergone previous FCD surgery with cortical resection. In two of the MDT‐positive FCDs, the signal changes were located predominantly in cortical areas. Previous studies have reported increased MD in white matter subjacent to FCDs and there are reports of gray and white matter MD alterations in FCDs that extend beyond the visible boundaries of the lesions.^15^, ^16^, ^17^ Multi‐compartment diffusion models have shown differences in diffusivity depending on cortical sampling depth according to FCD histological subtypes, where FCD IIa lesions predominantly show alterations in the superficial cortex, whereas FCD IIb lesions exhibit alterations that extend deeper into and below the cortex.^18^ MDT‐positivity is not specific, as increased FLAIR and MDT signals were also found in the resection margins of the postsurgical cases. In these cases, an increased MDT signal could be due to gliosis. The cause of the increased MD or MDT signals in FCDs remains unknown. Myelin has been shown to influence mean diffusivity, and in FCDs, there are reports of hypomyelination and a relative reduction of myelinated axons in the white matter beneath dysplasia, as well as aberrant myelin patterns in the cortex.^19^, ^20^ It remains unclear whether the MDT changes are related to the cause or consequence of epilepsy. MDT helped strengthen subtle or ambiguous findings in most FLAIR‐positive patients, and as such, MDT showed promise as a potential confirmatory tool. However, it remains unclear why MDT was more conspicuous than FLAIR in some FCD cases yet remained MDT‐negative in a few cases that were FLAIR‐positive. Further, it was clear that MDT could not identify lesions in patients previously deemed MRI‐negative with conventional MRI, and as such, MDT did not seem to be helpful in the management of this clinical group.

Interestingly, a prominent MDT intensity was observed in an FCD case where the MDT signal coincided with the hypermetabolic region seen in PET imaging (Figure 1C). The patient displayed very frequent focal non‐motor seizures daily, and we suggest that the MDT hyperintensity could possibly reflect peri‐ictal changes. Hypermetabolic PET signals have been associated with nonconvulsive status epilepticus, high spike frequency on EEG and may indicate the epileptogenic zone.^21^, ^22^ Another study reported co‐localization of PET hyperactivity, maximal neurophysiological activity on EEG and peri‐ictal lesions on MRI.^23^ Several reports have found diffusion changes in status epilepticus, where up to half of the cases showing MRI alterations.^24^ Most commonly, they have reported an increased DWI signal and decreased ADC signal.^25^ In experimental models of status epilepticus, reduced diffusion has been histologically associated with neuropil swelling in the acute phase, followed by increased diffusion corresponding to neuropil fragmentation.^26^ All of our FCD patients had a bright signal on the MDT maps, indicating increased diffusion. Possible explanations for the increased diffusion could include structural or functional changes in the tissue or vasogenic edema.

Another interesting finding in two of the FCD cases was a distinct disruption of the cortex on DWI obtained with high values (*b* = 2500 s/mm^2^). The disruption manifested as a reduced signal in the immediate cortical area of FCDs. To our knowledge, this finding has not been previously reported. The hypoattenuation is likely due to increased diffusivity in the cortex, which would be in line with our findings, where MDT shows increased cortical diffusion. However, another possible explanation could be increased susceptibility due to increased iron content, as this has been shown in FCDs.^27^

We found no MDT‐positive signal in heterotopias, and the juxtaposed subcortical tissue was MDT‐negative. Heterotopias have been demonstrated to have increased MD in perilesional white matter, which is not in line with our findings; however, these studies are based on quantitative assessment and are thus not directly comparable to ours.^28^, ^29^ The MDT maps are homogenous across tissue types and cannot distinguish white matter from gray matter. While MDT removes CSF‐related partial‐volume effects, it still doesn't automatically detect lesions whose inherent diffusion properties are similar to those of the surrounding tissue. Consequently, MDT could not delineate gray matter heterotopias.

As seizures are common in low‐grade gliomas (80–90%), we also wanted to explore the MDT sequence in these patients.^30^ There are reports of differences in MD values in low‐grade glioma patients related to seizures.^31^ Diffusion properties in low‐grade gliomas have been used for prognostic and diagnostic purposes.^32^ In low‐grade gliomas, the MDT signal correlated very well with the FLAIR signal, and MDT was more conspicuous than any other imaging protocol used. The delineation of low‐grade gliomas was surprisingly clear with MDT imaging and followed the FLAIR border very closely. However, we cannot determine whether MDT delineates the true tumor margin as we did not perform any histopathological sampling specifically targeted to assess this. MDT was also valuable in the early postoperative imaging, as ischemia appeared dark on MDT. New onset ischemia appears as an increased signal on FLAIR, making the distinction between FLAIR signals indicating tumor versus ischemia less evident compared with MDT. If future studies confirm these findings, MDT could be a valuable imaging modality both as a visual aid during surgical resections, for the immediate postoperative MRI evaluation of tumor remnants after resection, and perhaps for monitoring in the postoperative phase. A shared difficulty in assessing persistent tumor after glioma surgery is the radiological evaluation of possible FCD residuals after failed epilepsy surgery, as FCD remnants can also be difficult to discern from FLAIR‐positive postoperative changes. However, to investigate this further, it would necessitate early postoperative imaging after epilepsy surgery, as FLAIR changes induced by surgery unfold over time and then become difficult to discern from FCD.

## 5 LIMITATIONS

The key limitations of this study relate either to the study design or the MDT approach itself. The study design was exploratory, which may limit generalizability. However, the diversity of cases does demonstrate how the MDT responds to a variety of conditions. Analyses were qualitative and performed by a single neuroradiologist with access to clinical data, which introduced a potential observer bias. Another limitation is the lack of information on seizure activity immediately before the MDT exam, which is relevant, as seizures can induce transient alterations in FLAIR, T2, and diffusion imaging.^33^, ^34^ MDT was helpful in providing an improved visual delineation of the tumor‐normal tissue interface compared with FLAIR, but this improved delineation is not evidence of the true tumor infiltration margin, as we lack histopathological confirmation. The lesion visibility scoring system used in this study was intended for detection of lesions, not for evaluation of resection margins, and as such, it cannot provide information about the boundaries of abnormalities in the resection. Additionally, this study was not designed to compare how well MDT assesses the resection margin compared with sequences such as ADC maps or DWI, so MDT's performance relative to other modalities cannot be determined. The MDT approach also has its limitations. The technique is not specific, as MDT hyperintensities were observed both in probable FCD lesions and at resection margins, the latter likely reflecting gliosis. However, no MDT hyperintensity was present at the resection margins of the glioma case scanned early after surgery (<48 h). Specificity is particularly challenging in some regions, for example, adjacent to the frontal and posterior horns, where normal findings such as capping hyperintensities appear on both MDT and FLAIR, making potential lesions difficult to assess.^35^ Additional technical issues include low signal‐to‐noise ratios in iron‐rich structures such as the basal ganglia, fat‐related artifacts in some regions, and lengthy scan times.

## 6 FUTURE DIRECTIONS

Further studies are needed to address these limitations to validate our findings. Larger multicenter cohorts and blinded analyses with multiple readers are needed to assess inter‐observer reliability. Peri‐ictal assessments would be needed to clarify if there are functional contributions to the MDT signal. Histopathological correlations of lesions and tumor margins are needed to clarify the sensitivity and specificity of MDT. Further, comparisons to conventional modalities such as ADC and DWI are needed. Protocol optimization with improved SNR, mitigation of artifacts, faster acquisition time, and improvements in post‐processing to improve specificity would be needed to integrate MDT in the radiological workflow of tumor and epilepsy surgery.

## 7 CONCLUSION

MRI with MDT mapping is a complementary technique that facilitates visual assessment of radiological alterations in both epilepsy surgery and low‐grade glioma patients. Across cases, MDT increases the conspicuity of lesions relative to FLAIR in most epilepsy‐surgery cases, but not in MRI‐negative cases. MDT also improves visual demarcation of tumor‐associated signals compared with FLAIR in glioma patients. In FCD, increased MDT signal co‐localizes with the increased FLAIR signal in most cases and often appears more conspicuous, which suggests it may help to support subtle or inconspicuous FLAIR findings. MDT, in its current form, cannot be used as a visual screening tool for FCD, as it did not detect abnormalities in epilepsy surgery patients previously deemed MRI‐negative. Rather, MDT serves as an amplifier of alterations already visible on FLAIR. The MDT alterations could be either structural or functional, or perhaps both. In low‐grade gliomas, the MDT signal was more conspicuous and visually better demarcated than the FLAIR signal, suggesting potential utility in management and surgical planning. Taken together, while MDT in its current form cannot replace established techniques, it shows promise as a complementary tool. With further technical refinement, faster imaging protocols, and systematic evaluation, MDT may develop into a valuable imaging method in both epilepsy and tumor evaluations.

## AUTHOR CONTRIBUTIONS

**Irena Grubor:** data curation, formal analysis, investigation, methodology, project administration, visualization, validation, writing original draft preparation, review and editing. **Kristina Serdenicka:** formal analysis, investigation, methodology, validation, review and editing. **Cornelia Säll:** formal analysis, methodology, software, validation, review and editing. **Maria Compagno Strandberg:** investigation, methodology, validation, resources, review and editing. **Markus Nilsson:** conceptualization, data curation, formal analysis, investigation, methodology, resources, software, supervision, validation, visualization, review and editing. **Johan Bengzon:** conceptualization, investigation, methodology, resources, funding acquisition, supervision, validation, review and editing.

## CONFLICT OF INTEREST STATEMENT

MN has financial interests connected to b‐tensor encoding.

## ETHICS STATEMENT

The study was approved by the Swedish Ethical Review Authority (2022‐02803‐01). We confirm that we have read the Journal's position on issues involved in ethical publication and affirm that this report is consistent with those guidelines.

## Supporting information


**Figure S1.** MDT‐negative cases with findings on conventional MRI. Panel A shows periventricular heterotopia (white arrowhead) visible on T1‐weighted imaging. On MDT, the contour of the heterotopia is visible, but it is isointense with healthy tissue. MDT hyperintensities in the left frontal lobe correspond to the ulegyria displayed in Figure 2 in the manuscript. An asterisk indicates fat artifacts. Panel B demonstrates a hypothalamic hamartoma (white arrowhead) and Panel C shows polymicrogyria (white arrowhead) in the same patient, neither of which is clearly visible on MDT. Panels D‐F demonstrate suspected FCDs (white arrowheads) that are not visible on MDT. In Panel E, hyperintense regions on both FLAIR and MDT were found close to a previously resected area. Panel F shows a suspected bottom‐of‐sulcus dysplasia not visible on MDT.

Table S1.

## Data Availability

The data that support the findings of this study are available on request from the corresponding author. The data are not publicly available due to privacy or ethical restrictions.
