# Supplementary figures and images for: Microstructure imaging in patients undergoing evaluation for epilepsy surgery or low‐grade glioma: Clinical utility of a novel diffusion MRI method
Source: Epilepsia Open. 2026 Mar 6;11(2):592–604. doi: 10.1002/epi4.70244 (PMC13051824; doi:10.1002/epi4.70244)

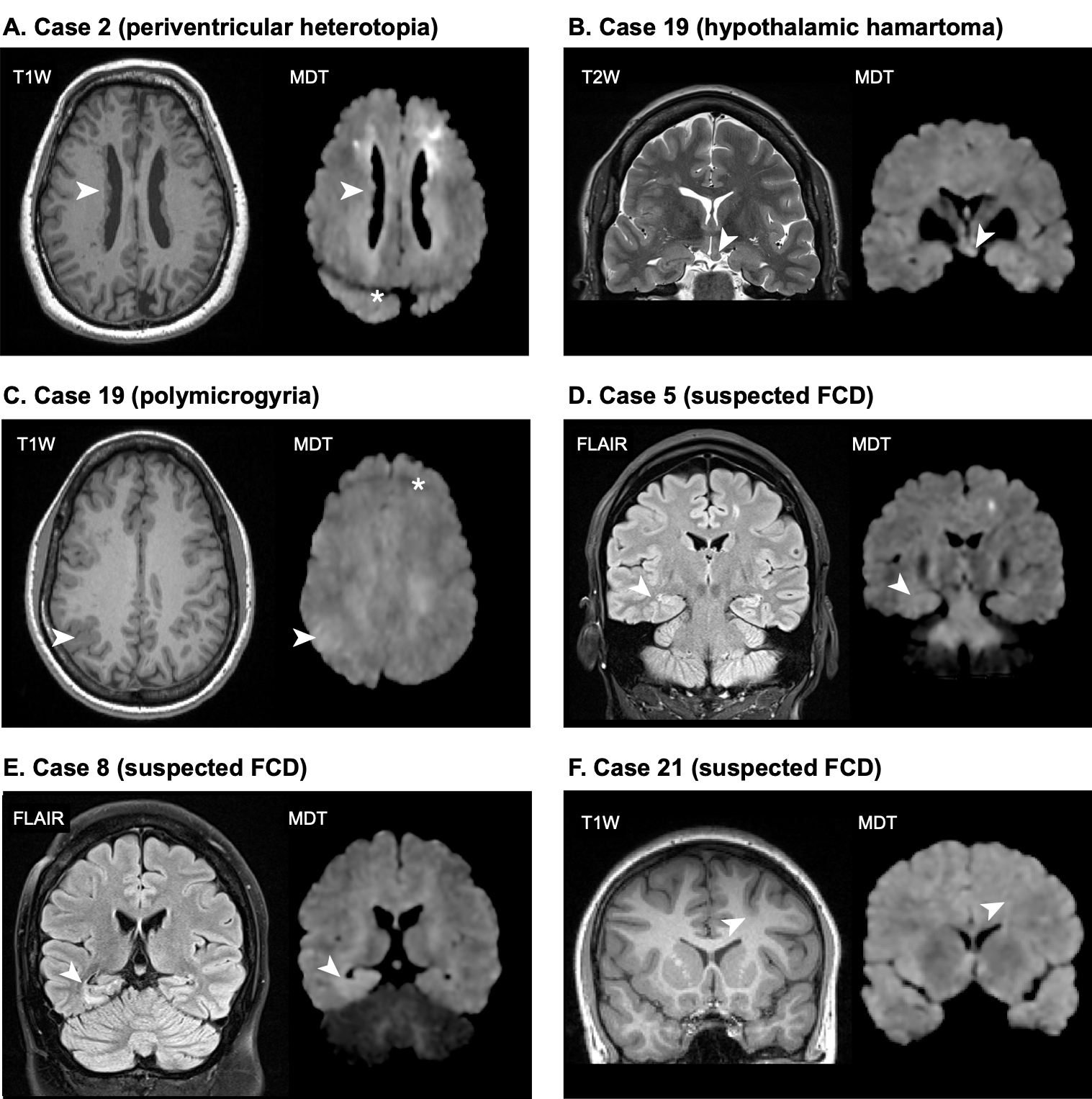

Supplement: Supplementary file 1 — Figure S1. MDT‐negative cases with findings on conventional MRI. Panel A shows periventricular heterotopia (white arrowhead) visible on T1‐weighted imaging. On MDT, the contour of the heterotopia is visible, but it is isointense with healthy tissue. MDT hyperintensities in the left frontal lobe correspond to the ulegyria displayed in Figure 2 in the manuscript. An asterisk indicates fat artifacts. Panel B demonstrates a hypothalamic hamartoma (white arrowhead) and Panel C shows polymicrogyria (white arrowhead) in the same patient, neither of which is clearly visible on MDT. Panels D‐F demonstrate suspected FCDs (white arrowheads) that are not visible on MDT. In Panel E, hyperintense regions on both FLAIR and MDT were found close to a previously resected area. Panel F shows a suspected bottom‐of‐sulcus dysplasia not visible on MDT. [file EPI4-11-592-s001.tiff]
