# Supplementary material for: Microstructure imaging in patients undergoing evaluation for epilepsy surgery or low‐grade glioma: Clinical utility of a novel diffusion MRI method
Source: Epilepsia Open. 2026 Mar 6;11(2):592–604. doi: 10.1002/epi4.70244 (PMC13051824; doi:10.1002/epi4.70244)
Supplement: Supplementary file 2 — Table S1. [file EPI4-11-592-s002.docx]

**Supplement 1. Clinical data of patients undergoing epilepsy surgery evaluation**

Abbreviations: FCD, focal cortical dysplasia; L, left; R, right; MRI-neg, MRI-negative; FIC, focal impaired consciousness seizure; FBTC, focal-to-bilateral-tonic-clonic-seizure; FPC, focal preserved consciousness seizure; BTC, unknown whether focal or generalized- bilateral-tonic-clonic-seizure; CSWS, Continuous Spike-and -Wave during Sleep; LTG, lamotrigine; BRV, brivaracetam; LEV, levetiracetam; LCM, lacosamide; CBZ, carbamazepine; OXC, oxcarbazepine, ZNS, zonisamide; CNB, cenobamate; PB, phenobarbital; CLB, clobazam; PER, perampanel; STM, sulthiame.

| Patient | Age | Age at seizure  onset | Sex | MRI finding | MDT | Seizure type | Seizure  Frequency  monthly | Anti-seizure medication |
| --- | --- | --- | --- | --- | --- | --- | --- | --- |
| 1 | 22 | 13 | F | FCD parietal L | + | FIC | 8 | LTG BRV |
| 2 | 36 | <1 | F | Ulegyria, frontal L  Subependymal heterotopias bilat | +  0 | FIC FBTC | 2 | LTG LEV |
| 3 | 46 | 26 | F | FCD remnant frontal L | + | FIC | 450 | LTG LEV LCM |
| 4 | 31 | <10 | M | FCD insula R | + | FPC | 214 | CBZ LEV |
| 5 | 40 | 15 | F | FCD temporal R | 0 | FIC | 2 | LTG |
| 6 | 32 | 4 | F | MRI-neg | 0 | FIC FBTC | 90 | LTG OXC |
| 7 | 55 | 21 | M | Subependymal heterotopias bilat | 0 | FIC FBTC | 0.3 | CBZ BRV |
| 8 | 33 | <1 | F | FCD temporal R  FCD remnant parietal R | 0  + | FIC | 18 | LEV OXC ZNS |
| 9 | 25 | 8 | M | MRI-neg | 0 | FPC FBTC | 25 | OXC CNB LCM |
| 10 | 43 | 29 | M | MRI-neg | 0 | FIC | 15 | BRV PB CLB PER |
| 11 | 37 | 34 | F | MRI-neg | 0 | BTC | 1 | LEV LCM |
| 12 | 10 | 5 | F | Transmantle frontal R | + | FIC | CSWS | LTG VPA STM |
| 13 | 48 | 44 | F | MRI-neg | 0 | FIC | 24 | LTG LEV |
| 14 | 14 | 6 | F | MRI-neg | 0 | FIC | 1 | LTG OXC STM |
| 18 | 21 | 14 | F | MRI-neg | 0 | FIC | 34 | LTG LEV LCM CNB |
| 19 | 45 | 11 | F | Polymicrogyria, R frontal. Hypothalamic hamartoma | 0  0 | FIC | 32 | LTG BRV LCM |
| 21 | 17 | 2 | F | FCD frontal R | 0 | FIC | 4 | LTG |
| 22 | 19 | 9 | M | MRI-neg | 0 | FIC | 31 | LTG LEV CLB |
